# Neuronal Morphology and Synapse Count in the Nematode Worm

**DOI:** 10.3389/fncom.2019.00074

**Published:** 2019-10-22

**Authors:** Robert Friedman

**Affiliations:** Department of Biological Sciences, University of South Carolina, Columbia, SC, United States

**Keywords:** neuronal morphology, nematode worm, synapse count, neuronal anatomy, *Caenorhabditis elegans*

## Abstract

The somatic nervous system of the nematode worm *Caenorhabditis elegans* is a model for understanding the physical characteristics of the neurons and their interconnections. Its neurons show high variation in morphological attributes. This study investigates the relationship of neuronal morphology to the number of synapses per neuron. Morphology is also examined for any detectable association with neuron cell type or ganglion membership.

## Introduction

The nervous system, including the synaptic connections which link a neuron to a target cell, has evolved more than once in animals (Ryan and Grant, 2009; Emes and Grant, 2012; Dunn et al., 2015; Moroz and Kohn, 2016). Both the neuron and the synapse have many types and shapes (Ramon y Cajal, 1899; Masland, 2004; Ascoli, 2006; Halavi et al., 2012; Gouwens et al., 2019) which function in the processing of information (Schwartz and Simoncelli, 2001; Hickok and Poeppel, 2007; Emes et al., 2008; Bullmore and Sporns, 2012). This system has been modified along a path that is constrained by biological evolution (Waddington, 1942; Elston et al., 2001; Laughlin and Sejnowski, 2003; Elston, 2007; Elston and Manger, 2014; Moroz and Kohn, 2016; Niven and Chittka, 2016; Burkhardt and Sprecher, 2017), cellular processes which include stochastic mechanisms (Nicklas, 1997; Kaern et al., 2005; Cai et al., 2006; Faisal et al., 2008; Almog and Korngreen, 2016), and physical constraint of cellular structure in tissue (Ramon y Cajal, 1899; Elston, 2007; Lecuit and Lenne, 2007).

The nematode worm *Caenorhabditis elegans* is a relatively simple model for understanding neuronal connections, synaptic processes, and the relationship between the neural system and animal behavior (Ward et al., 1975; Chklovskii, 2004; Gray et al., 2005; Schafer, 2016; Karbowski, 2018). The adult hermaphroditic form has two distinct nervous systems, one somatic with 282 neurons (White et al., 1986) and a second pharyngeal system with 20 neurons (Albertson and Thomson, 1976). These neurons are overconnected as a network and likewise each neuron is expected to chart a path of three or less synapses to any other neuron in the system (Bargmann, 2012; Yan et al., 2017).

However, typical use of the neuronal network is constrained and regulated by the mechanism of neuronal modulation (Nadim and Bucher, 2014). Another constraint is the finding that this network in *C. elegans* favors certain motifs in the case of neurons with five or more synapses. These motifs include the feed-forward loop, bi-fan, and bi-parallel motifs (Milo et al., 2002). These motifs are indicative of the building blocks of a *C. elegans* neural circuit and therefore a fundamental unit in the explanation of information processing, input of sensory information, and output for motor control of the animal.

**FIGURE 1:**
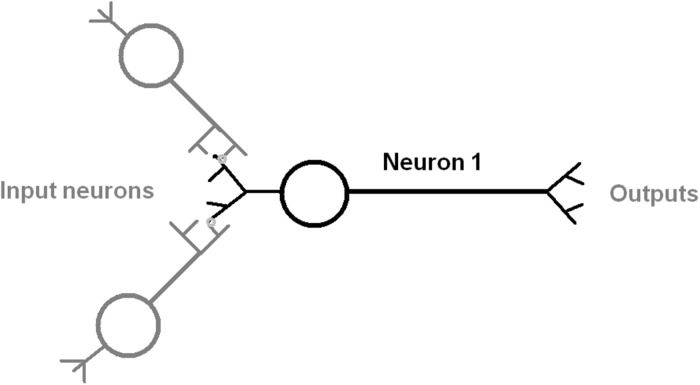
Diagram of an idealized neuron with multiple inputs and outputs that indicate the direction of information flow. The input process is shown as mediated by a synapse between dendrite and axon of an input neuron (gray color). The output is by an axonal process to another neuron.

The anatomy of a typical neuron includes the soma cell body size, the number of dendrites, branches from the axon, and axon length (Ascoli, 2006; Scorcioni et al., 2008; Figure 1). The web database at http://NeuroMorpho.Org includes curated records of digitally reconstructed neuron structure and their morphological measurements across many animal species (Meijering, 2010; Donohue and Ascoli, 2011; Feng et al., 2015). This data was analyzed by principal component analysis (PCA) to further our understanding of the distinctiveness of neuronal morphology in *C. elegans* (Costa Lda et al., 2010; Guerra et al., 2011; Hobert et al., 2016) and also its relationship to the number of synaptic connections per neuron (Chklovskii, 2004; Elston, 2007; Rees et al., 2017).

## Methods

### Data Retrieval

http://NeuroMorpho.Org (Ascoli, 2006; accessed August 2019) was the source of morphological data of neurons in the following metazoan animal species: *C. elegans* (*N* = 603), the fruit fly (*N* = 28484), and rat (*N* = 27520). This database was accessed by its web based interface and the use of a first step in an informatics pipeline which consisted of shell based scripts and the command-line software *curl*.

The cell type of each neuron in *C. elegans* was annotated from an associated publication source whenever available (see http://NeuroMorpho.Org). The ganglion type, synaptic count, and span length associated with each neuron in *C. elegans* was listed in a data table at https://www.wormatlas.org/ (Varshney et al., 2011).

### Data Preprocessing

**FIGURE 2:**
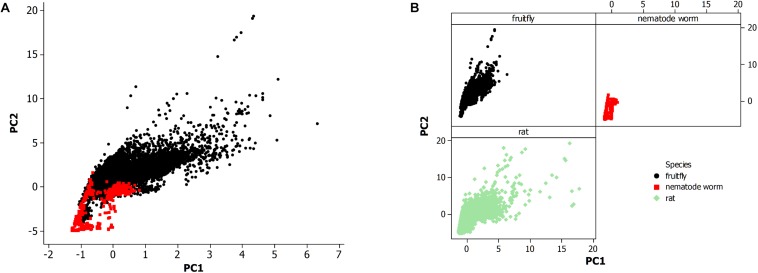
**(A)** Principal component analysis of neuronal morphological data in the nervous system of the fruit fly (*N* = 28,355) and all life stages of the non-pharyngeal regions of the nematode worm (*N* = 580). PC1, principal component 1; PC2, principal component 2. **(B)** Principal component analysis from panel **(A)** where each species is displayed in a separate panel. Also, included the analysis of all available neurons in rat (*N* = 27,426).

The second step of the pipeline used a combination of shell and Perl scripts to parse the database records in tabular format. This step retained only the non-redundant set of records that corresponded to the three species listed above. This was the data set for Figure 2. For *C. elegans*, records were then associated with an annotated ganglion type, neuronal cell type, a life history stage, and further verified for correctness. The final set of neuronal data for the adult hermaphroditic form consisted of 265 neurons out of the total possible set of 280 non-pharyngeal neurons with one or more synaptic connections.

### Data Processing

Minitab statistical software (version 14; Wild, 2005) was used for further data organization, grouping by category, and testing by PCA. The sample size of the resultant data sets is reported in the results. Also, excluded 29 neuron records of rat from the PCA scatterplot in Figure 2B since the principal component values were high (PC1 > 40; PC2 > 20) which led to outlying data points and an overly compressed data plot.

The PCA was based on a correlation matrix since the morphological data are not measured in the same type of unit. A Scree plot provided a diagnostic to measure the data variability that is captured by each of the principal components and also to assess the optimal choice of components for data exploration. This data contains each of these 16 morphological features (http://NeuroMorpho.Org; Ascoli, 2006; Scorcioni et al., 2008; Hobert et al., 2016): surface, volume, fractal dimension (Elston, 2007), neuronal width, neuronal height, neuronal depth, node diameter, Euclidean distance from soma, path distance from soma, branch contraction, branch fragmentation, soma surface area, number of neuronal stems, number of neuronal bifurcations, number of neuronal branches, and branch length. Apart from the neuronal measurements and the number of features, many of the features are a summary statistic because it allows for multiple features per neuron.

## Results and Discussion

### Comparison of Neuronal Morphology in Animals

The morphological data of the nematode worm *C. elegans* is compared to other metazoan animals, the fruit fly of insects, and the rat of mammals (Figure 2). Figure 2A shows a PCA of the nematode worm with the fruit fly, with the rat excluded to more easily observe the overlapping of data points among the animal species. Principal components 1 and 2 (PC1 and PC2) were plotted since they include the majority of the variation in the morphological data set while the alternative principal components, PC3 and PC4, did not produce a substantially different result. Therefore, the overlap in the data points between the nematode worm and fruit fly is from similarity of neuronal morphology.

The fruit fly has a much larger population of neurons than the nematode worm and likewise the plot shows that the fruit fly has greater neuronal morphological variation as shown by data points at higher values along both axes. The rat and fruit fly comparison is shown in Figure 2B, indicating further overlap of morphological measurements among different species, but with rat showing yet greater morphological variation than the fruit fly.

The nematode worm has a much simpler nervous system as compared to the other species in this study, so simplicity could result in fewer neuronal morphologies. A second possibility is that neurons vary not altogether by complexity of the system but instead by the economy of space within the neural tissue. Further, the review by Elston (2007) proposed a multifactor cause of neuronal morphological differences among species, a result of studies of pyramidal cells in the cerebral cortex, including the visual and prefrontal regions, of primates. This finding is further supported by a study of the visual cortex in rodents and primates (Elston and Manger, 2014).

### Neuronal Morphology as a Predictor of Cell Type or Location

**FIGURE 3:**
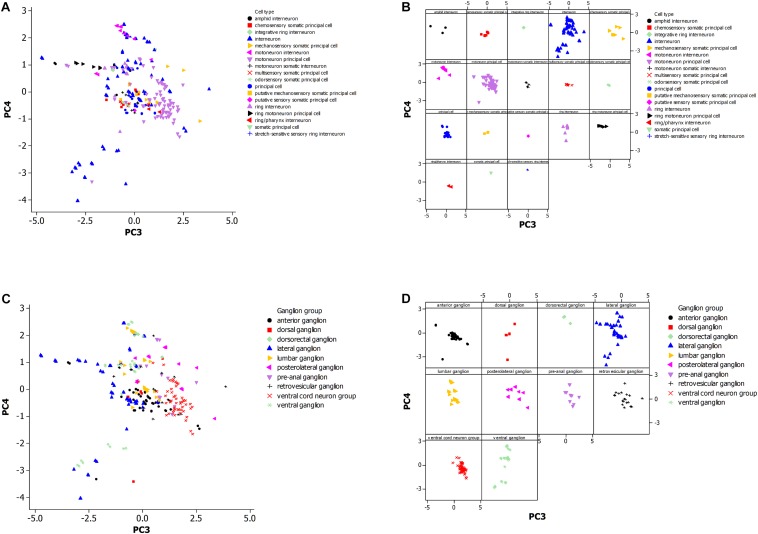
**(A)** Principal component analysis of neuronal morphological data in the non-pharyngeal regions of the adult nematode worm (*N* = 265). PC3, principal component 3; PC4, principal component 4. Grouped data by cell type. **(B)** Same plot as panel **(A)** where each cell type is displayed in a separate panel. **(C)** Same plot as panel **(A)** but instead data was grouped by ganglion type. **(D)** Same plot as panel **(C)** where each ganglion type is displayed in a separate panel.

Figure 3 shows plots of the principal components (PC values) of the neuronal morphological data for *C. elegans*. This PCA analysis was employed as an exploratory statistical method and the use of PC3 and PC4 provided candidate groupings to classify neurons by cell type or by ganglion membership.

Figure 3A shows the PCA plot (Figure 3B is the same plot except for separate panels for better visualization of the data). The tighter the grouping by morphology for a cell type (Figure 3B), and the greater the non-overlap of the group with others, the more reliable the use of the morphological data in establishing a cell type group as distinctive. The motoneuron principal and ring motoneuron principal neurons of *C. elegans* are candidates for this definition of distinctiveness. Others are difficult to suggest the same because of small sample size or coincidence with the morphology of another group.

Figure 3C shows distinctiveness for some groups of neurons that are grouped by ganglion type. These groups may cluster in the plot because of constraint on neuronal morphology within a ganglion or by constraints at a larger scale such as optimization for neuronal connectiveness. The anterior and retrovesicular ganglia both show a tight grouping of associated neurons (Figure 3D), but their overlap in Figure 3C prevents their assignment to distinctive groups. The ventral cord neuron group appears as the best candidate for a distinctive group. Given the differences in the sample size of the neuronal population by cell type and ganglia, it is difficult to compare whether morphological features of the neurons are a better predictor of cell type or ganglion membership.

**FIGURE 4:**
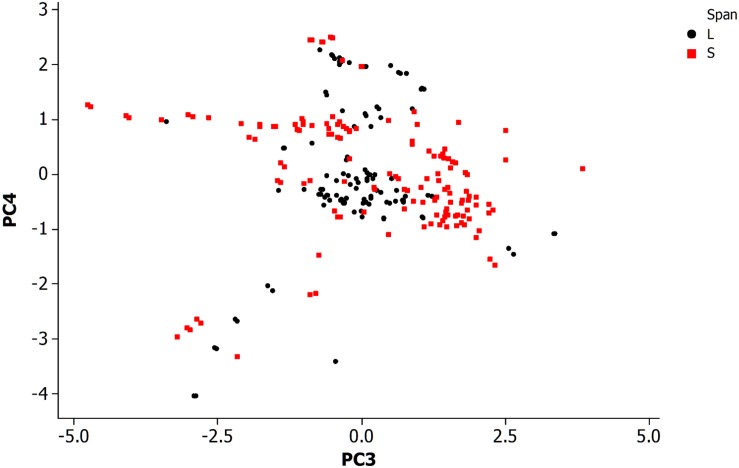
Principal component analysis of neuronal morphological data in the non-pharyngeal regions of the adult nematode worm (*N* = 265). PC3, principal component 3; PC4, principal component 4. Grouped data by length of neuronal span: a neuronal span of less than 25% of the body length are described as a short span (S) while all others are long span (L).

Figure 4 is a PCA plot (PC3 and PC4) of neurons categorized by span length across the animal. The definitions for long and short spanning neurons were established at https://www.wormatlas.org/ where short span are defined as neurons with processes confined to less than 25% of the worm body while the remainder are defined as long spanning. Most of the long spanning neurons appear reasonably distinctive from the other short spanning type. This result has limited application for making predictions about neurons, but does provide insight into the organization of this simple somatic nervous system.

The above results show that a study based on a sample of a population of neurons is problematic for identification of distinct groups, such as by morphology, since further sampling from the population and its variability across features is expected to lead to loss of distinctiveness. This may also occur by chance given an animal has a small population of neurons, a confounding factor in any correlation analysis.

### Neuronal Morphology as a Predictor of Synaptic Connections

**FIGURE 5:**
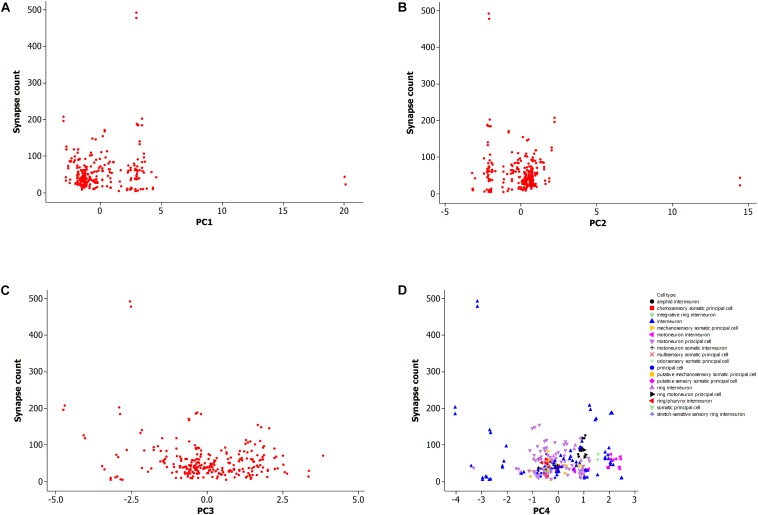
**(A)** Plot of synapse count versus principal component 1. Data sample same as in Figures 2, 3. **(B)** Plot of synapse count versus principal component 2. **(C)** Plot of synapse count versus principal component 3. **(D)** Plot of synapse count versus principal component 4 and grouped data by cell type.

Figure 5 shows a data plot of the first four principal components which represent neuron morphology against the number of synaptic connections. All panels, Figures 5A–D, do not show clear association between neuronal morphology and synaptic count. This data includes both types of synapses, electrical and chemical, including the subtype for neuromuscular junctions. Figure 5D further shows that morphology per cell type is not related to synapse count. However, it does identify two outlying data points with high synapse count as an interneuron cell type.

This result suggests that a large scale analysis of neuronal morphology in *C. elegans*, including number of neuronal branches and the overall size of the neuron, is not predictive of the number of synaptic connections to other cells. It is also not supportive of the hypothesis that the neuronal morphological measurements in this study are sufficient to explain the potentiality of neurons to form synapses with other cells.

It has been shown that neurons in *C. elegans* are greatly overconnected (Varshney et al., 2011) and that the potential number of paths in the neuronal network is not fully explored at a given point in time and for a given environment, but instead that most network paths are latent (Chen et al., 2006; Bargmann, 2012; Ganguli and Sompolinsky, 2012). In addition, it is possible that some of the paths are rarely used or not active over the life history of the animal. However, the activity along neuronal paths, neural circuits (Milo et al., 2002), and their functions, are generally not yet well known apart from some examples (Gray et al., 2005; Bargmann, 2012; Borst and Helmstaedter, 2015). It is possible that a highly connected neuron uses just a few out of a large number of potential connections while another neuron has few synaptic connections that are continually active. In this latter case, knowledge of the synapse itself (Ryan and Grant, 2009; Emes and Grant, 2012) is considered necessary in predicting neuronal activity while other factors such as morphology are less informative. This is suggested from this large scale analysis of a neuronal population.

One approach toward understanding synaptic function is from the theory of dynamical systems (Spruston, 2008; Hu et al., 2013). This includes knowledge for modeling the electrical and physiological properties of a neuronal cell and its reliability in communicating information across synaptic connections. For example, it is expected that the number of synapses for a neuron is correlated with its handling of signal originating from multiple sources, such as from input neurons (Han et al., 2017; Han et al., 2018; Ma et al., 2019). This suggests that the higher scales of signal processing is a predictor of synaptic count.

Studies in primates and rodents support this view (Elston et al., 2001; DeFelipe et al., 2002; Benavides-Piccione et al., 2006; Spruston, 2008; Elston and Manger, 2014). Previous work has found a correlation between the complexity of the dendrite and dendritic spines of primate cortical cells and their role in the hierarchy of information processing (Elston et al., 2001; Jacobs and Scheibel, 2002; Elston, 2007). Elston (2003) found a similar result in New World and Old World monkeys by comparing pyramidal cells between the prefrontal cortex and primary visual area. Further support for neuronal morphology as an indicator of cortical function is reviewed by DeFelipe et al. (2013), Luebke (2017), and an in depth reference by the Petilla Interneuron Nomenclature Group (2008).

However, the above patterns of neuronal diversity are complex and not necessarily generalizable across mammalian orders (Elston et al., 2001; Elston and Manger, 2014). Overall, these findings support that the cerebral cortex of a mammal is not built of a single building block neuronal cell, but instead by a diverse set of neuronal cell types as defined by their physical properties. Elston et al. (2001) further showed that the advanced information processing among higher primates is not merely achieved by evolving a larger number of building block neurons, but instead that pyramidal cell morphology and dendritic structure is likely adapted by cortical region and its neural circuitry. These known patterns of neuronal organization in mammals provide a context for interpretation of the findings of this study.

## Data Availability Statement

Publicly available datasets were analyzed in this study. This data can be found here: http://neuromorpho.org/ and https://www.wormatlas.org/.

## Author Contributions

The author confirms being the sole contributor of this work and has approved it for publication.

## Conflict of Interest

The author declares that the research was conducted in the absence of any commercial or financial relationships that could be construed as a potential conflict of interest.
